# A Broad Wildlife Survey of Influenza A Virus in the Orinoco Flooded Savannas from Colombia: New Reports and Perspectives

**DOI:** 10.3390/ani15152201

**Published:** 2025-07-26

**Authors:** Astrid Katerine Cárdenas Parra, Juan Pablo Barón Vera, Iván Fernando Calixto-Botía, Nubia E. Matta, Oscar Andrés Rodríguez-Fandiño, Lady Johana Correa-Higuera

**Affiliations:** 1 Laboratorio de Estudios Moleculares de la Orinoquia, Universidad Internacional del Trópico Americano (Unitrópico), Yopal 850002, Colombia; astridcardenas@unitropico.edu.co (A.K.C.P.); patc@unitropico.edu.co (J.P.B.V.); pe.lemo@unitropico.edu.co (I.F.C.-B.); 2 Laboratorio Relación Parásito Hospedero, Departamento de Biología, Facultad de Ciencias, Universidad Nacional de Colombia, Bogotá 111321, Colombia; nemattac@unal.edu.co

**Keywords:** IAV, wildlife, Serology, qPCR, surveillance, Orinoquia, zoonotic

## Abstract

Influenza A virus (IAV) poses a significant threat to animal and public health due to its wide host range and potential for interspecies transmission. This study aimed to conduct a comprehensive survey of IAV in a wide range of wildlife in the Orinoco flooded savannas of Colombia, a region of high biodiversity and a strategic location for monitoring viral transmission. Sampling was conducted during both dry and rainy seasons in two localities. ELISA and qPCR targeted 2028 individuals from 173 bird, mammal, and reptile species, 124 of them without previously published reports. There were positive results for 54 species, with 34 representing first-time world reports. Of the qPCR, 13.4% were positive from birds, and 2.9% were from mammals. Seropositivity was identified in 5.7% of birds, 2.7% of mammals, and 1.3% of reptiles. These findings underscore the potential role of these diverse species as reservoirs or incidental hosts in the transmission cycle of IAV, emphasizing the need for expanded research on less-studied taxa and their ecological interactions. The results also contribute to our understanding of the epidemiology of IAV in the Neotropics and can inform future surveillance and mitigation strategies.

## Simple Summary

The Orinoquia region, located in northern South America, is a biodiverse ecosystem characterized by extensive floodable savannas that serve as a migratory stop-over for numerous species of birds, some of which act as natural reservoirs for zoonotic pathogens such as influenza A virus, an infectious agent that can be fatal in humans and other species. Reducing the impacts caused by avian influenza requires constant monitoring of the virus, including its geographic distribution and host types. In this study, we conducted a comprehensive survey of the virus in wildlife and livestock animals using serological and molecular techniques. We detected prior exposures and the active presence of the virus in migratory aquatic birds. Notably, we discovered new global reports in birds, mammals, and reptiles that are part of wildlife, such as capybara and caiman. Additionally, we found interesting positive results in livestock, including a new global report on domestic sheep. These findings underscore the need to implement monitoring and surveillance strategies for the virus in the Orinoquia region.

## 1. Introduction

The flooded savannas of the Orinoquia in Colombia are distinguished by their ecological, economic, and cultural significance. These wetlands provide essential natural resources for national consumption and are crucial for the conservation of numerous species and reinforce the cultural identity of the local human communities. Primarily composed of seasonally flooded savannas, the Orinoquia wetlands feature a dynamic landscape influenced by the seasonality of precipitation [1,2]. From October to February, the region experiences dry conditions, creating an arid environment that concentrates wildlife around small water bodies, and, at the same time, it becomes the season for migrants. In contrast, during the rainy season, the area transforms into an extensive network of aquatic environments, including both lotic and lentic systems, which are either temporary or permanent. This transformation facilitates extensive movement of the fauna that inhabit these wetlands [3,4].

The biodiversity of this region is particularly remarkable, especially concerning avian species [5,6,7]. From the approximately 1966 species of birds recorded in Colombia [8], the savannas from Orinoquia are home to more than 761 bird species [9] and serve as vital stop-over points for migratory birds with routes originating from North America between October and March and from the southern latitudes from April to September, establishing ecological connectivity between this region and the rest of the continent [10,11,12]. In addition, bird migrations are not confined to large-scale continental movements, but many resident species potentially engage in altitudinal migrations or movements on a regional scale. The Orinoquia is also home to a rich diversity of mammals, including species adapted to open savannas, gallery forests, and aquatic ecosystems [13], such as the giant anteater (*Myrmecophaga tridactyla*) and the capybara (*Hydrochoerus hydrochaeris*), which play key roles in the ecological dynamics of the region [13]. Iconic predators like the jaguar (*Panthera onca*) also inhabit the area, illustrating the region’s status as a biodiversity hotspot. The reptile fauna of Orinoquia is equally diverse, with species such as the Orinoco crocodile (*Crocodylus intermedius*), a critically endangered species [14], the common caiman (*Caiman crocodilus*), the savanna side-necked turtle (*Podocnemis vogli*) and the mata-mata (*Chelus fimbriata*), further showcasing the ecological diversity and evolutionary adaptations within the region.

This biological dynamism underscores the need for continuous monitoring to understand the movement patterns of these species and assess associated health risks, such as the presence of Influenza A virus (IAV), the primary agent responsible for avian influenza outbreaks, which has gained significant importance in recent times [15,16,17]. IAV represents a major threat to animal health and possesses an ongoing zoonotic risk due to its ability to infect a wide range of hosts [18], primarily in avian, the main reservoir, as well as mammalian species [16]. Sporadic detections have been reported in reptiles and, seemingly, in amphibians [19,20,21,22,23,24,25], reflecting a limited understanding of host ranges, viral maintenance and transmission dynamics.

IAV belongs to the Orthomyxoviridae family and is an enveloped, single-stranded, negative-sense RNA virus. It is classified into types based on differences in their matrix and nucleoprotein antigens, with type A being the most significant regarding human health [26]. IAV is further divided into subtypes based on surface glycoproteins, hemagglutinin (H) and neuraminidase (N), which play critical roles in the virus’s ability to infect cells and evade the immune response [27,28,29]. To date, at least 16 H subtypes and 9 N subtypes have been identified in the avian species, within waterfowl and shorebirds (Anseriformes and Charadriiformes) [15,30]. Recent studies have expanded this number, identifying the H19 subtype in aquatic birds [31,32]. In contrast, the H17 and H18 subtypes, along with N10 and N11, have been detected in bats, indicating a difference in the distribution of these subtypes according to the host group [33].

The epidemiological impact of IAV is exacerbated by its ability to cross species barriers, leading to the emergence of new outbreaks [17]. Particularly relevant are the recent records of avian influenza movements observed in various regions of South America, especially those along the coastal areas of Chile and Peru [30,34,35,36]. These findings underscore the critical role of migratory birds in the long-distance dissemination of this pathogen in South America, emphasizing the need for strengthened surveillance efforts.

In this context, the present research aimed to conduct a comprehensive survey of Influenza A virus in the Orinoquia region of the department of Casanare, Colombia, as a strategic bioregion for monitoring the transmission dynamics of IAV among wildlife populations. For this purpose, we covered the dry and rainy seasons in two localities with differing levels of anthropic disturbance in 2023–2024. We employed a serological approach using ELISA and molecular detection via qPCR, targeting a wide range of bird, mammal, and reptile species, including wildlife used as bushmeat and livestock. Special emphasis on sampling was placed on aquatic and semi-aquatic habitats, particularly those associated with anatids, a group of birds recognized as primary hosts for the maintenance and spread of Influenza A virus [15]. This study contributes to understanding the ecology of influenza A virus in the Orinoquia’s wetlands, providing strategic knowledge for developing effective conservation and public health measures and highlighting the importance of coordinated surveillance and response strategies to mitigate the spillover risks of Influenza A virus.

## 2. Materials and Methods

### 2.1. Study Area Definition

**Figure 1:**
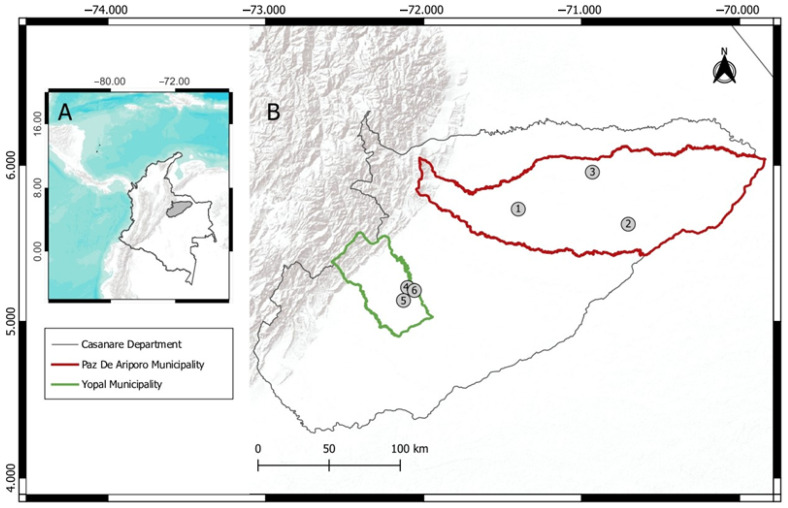
The geographical location of sampling sites: (**A**) Casanare’s location in Colombia; (**B**) Department of Casanare; (Red) Municipality of Paz de Ariporo and sampling sites: (1) Locality of RNSC “Aves D’ JAH” (05°43′946″ N, 71°24′451″ W), (2) AICA (Important Bird Areas) “Chaviripa—El Rubí” (5°37′22.5″ N, 70°42′18.5″ W), (3) Farm “La Carpeta” (5°57′21.00″ N, 70°55′55.30″ W); (Green) Municipality of El Yopal and sampling sites: (4) Farm “La Llave” (5°13′47.8″ N, 72°6′74.5″ W), (5) Farm “Buenas Tardes—Matapalo” (5°8′7.4.4″ N, 72°7′42.2″ W), and (6) Ranch “La Virgen” (5°11′55.90″ N, 72°3′33.40″ W).

This study was conducted in the department of Casanare, Colombia (Figure 1) and involved capturing and sampling wildlife (birds, mammals and reptiles), including some livestock. Casanare consists mainly of the flooded savannas of the Orinoquia, with a mainly flat topography dominated by seasonal water bodies such as morichals (a wetland ecosystem dominated by the palm tree *Mauritia flexuosa*) that are affected by the rainy season [37]. Sampling was carried out in two municipalities (Figure 1, Table 1): (1) Paz de Ariporo, characterized by a combination of floodable savannas and wetlands; due to its large size and low population density, this municipality contains areas of protected native flora and fauna, despite frequent interventions related to agriculture, extensive cattle ranching, and oil exploitation. (2) El Yopal, considered a transitional region between the foothills of the plains and the floodable savanna; has a higher degree of anthropic intervention and is close to the department’s capital. Surveys were conducted in June 2023 (rainy season), March 2024 (dry season) and June 2024 (rainy season) in each of the two municipalities, for a total of six sites.

### 2.2. Sample Collection

Oropharyngeal, cloacal/rectal swabs were collected using sterile flocked nylon swabs per individual, which were stored separately in DNA/RNA Shield (ZYMO) until processing. Blood samples were also collected and centrifuged at 10,000 rpm for 10 min to obtain serum and stored at 4 °C until processing. For smaller species, especially birds of the order Passeriformes, blood samples were pooled in groups of 4–5 individuals, depending on the species. The samples were transported in a cold chain to the laboratory and stored at −80 °C until analysis. Two complementary techniques were used to get a perspective of IAV circulation in the region at different stages of infection: ELISA, for detection of previous exposures to the virus, and qPCR for detection of current viral load in the organism.

### 2.3. Molecular Detection of Influenza A Virus

IAV detection was performed by real-time multiplex qPCR. Primers and probe for all influenza A virus lineages were synthesized according to Spackman et al. [38] as follows: forward (M +25) 5′-AGATGAGTCTTCTAACCGAGGTCG-3′, reverse (M-124) 5′-TGCAAAAACATCTTCAAGTCTCTG-3′, probe 5′-FAM-TCAGGCCCCCTCAAAGCCGA-BHQ1-3′. The 18S rRNA gene of the host was used as internal control following Zyrianova and Zaripov [39]: forward 5′-GAGCTAATACATACATGCCGCCGACGAG-3′, reverse 5′-CTAGAGTCACCAAAGCTGCC-3′, probe 5′-HEX-CGACCTCCCCGGGGGGGACG-BHQ1-3′. The Luna^®^ Universal Probe One-Step RT-qPCR Kit (NEB) was performed for the assay, with a reaction mixture containing 10 µL Luna reaction mix, 1 µL Luna enzyme mix, 0.5 µM IAV primers, 0.3 µM probes and 18S primer, and 5 µL of RNA extract, in a final volume of 15 µL. Amplification conditions consisted of an initial reverse transcription step at 55 °C for 10 min, DNA denaturation at 95 °C for 10 min, and 40 cycles of 15 s at 95 °C and 1 min at 60 °C for hybridization and extension. Samples with a CT for 18S < 35 and a CT for M < 40 were considered positive.

### 2.4. Detection of Antibodies Against Influenza A Virus

Two commercial kits were used to detect antibodies to IAV: the blocking kit IDEXX AI Multi S-Screen Ab ELISA test (IDEXX Laboratories, Westbrook, ME, USA) for avian samples and the ID Screen^®^ Influenza A Antibody Competition Multi-species (Innovative Diagnostics, Grabels, Hérault, France) for mammalian and reptile samples. Since the kit lacks reptile validation, it was deemed experimental and employed as a preliminary tool to assess IAV exposure in this taxonomic group. These samples were processed according to the manufacturer’s instructions using the positive and negative controls provided with the kit.

**Figure 2:**
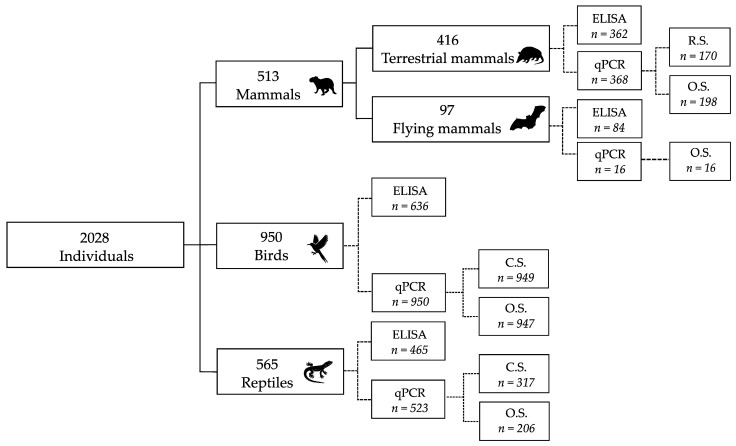
Processing by taxonomic group, analytical technique and matrix tested. Of the 2028 screened individuals, some were tested using both analytical techniques (ELISA and qPCR) and, in the case of qPCR, by more than one matrix (O.S., C.S. and R.S.), summing 3404 samples in total, 1857 samples by qPCR and 1547 samples by ELISA. n = Number of samples processed, O.S. = Oropharyngeal swab, C.S. = Cloacal swab, R.S. = Rectal swab.

Figure 2 shows the distribution of processed samples by taxonomic group and analytical technique.

### 2.5. Search for New Host Reports

To identify new reports of first-time hosts investigated for IAV, we used the PUBMED platform and the following search equation “Avian influenza” AND (Cuba OR Colombia OR Peru OR Brazil OR Ecuador OR Venezuela OR Chile OR Neotropic OR Guyana OR Suriname OR ‘French Guyana’ OR Costa Rica OR Belize OR Honduras OR Panama OR Nicaragua OR Salvador OR Argentina OR Bolivia OR Uruguay OR Paraguay OR ‘Lesser Antilles’ OR ‘Great Antilles’) AND (birds OR reptiles OR crocodiles OR mammals)” as well as a search in the google scholar database using as a search engine: “Scientific name” AND ‘Influenza’ and ‘English name’ AND ‘influenza’ and the latest version of the “Global Avian Influenza Viruses with Zoonotic Potential situation update” updated on 25 December 2024 [40].

## 3. Results

A total of 2028 individuals were screened during the fieldwork, distributed over 173 species of the three taxonomic groups studied (Table 1; Figure 2), providing a remarkable sweep of wildlife diversity to detect the presence and circulation of Influenza A virus in the Colombian Orinoco. Birds represented the group with the highest number of captures across the three sampling periods, with 950 individuals from 125 species, including representatives of the area such as the wattled jacana (*Jacana jacana*), the southern lapwing (*Vanellus cayanensis*), the orinoco goose (*Oressochen jubatus*) as well as some migratory species such as the blue-winged teal (*Spatula discors*) and the least sandpiper (*Calidris minutilla*). Additionally, a significant number of birds belong to the order Passeriformes. Among the mammals, 513 individuals from 42 species were captured, including wild species common in the Colombian Orinoco, such as the armadillo (*Dasypus sabanicola*), the giant anteater (*Myrmecophaga tridactyla*), and the capybara (*Hydrochoerus hydrochaeris*), as well as some domestic species common in the area, such as cattle (*Bos taurus*), domestic sheep (*Ovis orientalis aries domestica*) and wild swine (*Sus scrofa*). Finally, in the reptile group, 565 individuals from six species were captured, mainly represented by the green iguana (*Iguana iguana*), the common caiman (*Caiman crocodilus*) and the savanna side-necked turtle (*Podocnemis vogli*).

Our results demonstrated virus circulation, with positive cases detected across all three taxonomic groups, including both wildlife and livestock animals commonly found in the study areas. For some of the 2028 individuals, it was possible to obtain more than one matrix, either to test the two analytical techniques or, in the case of qPCR, to screen two matrices (oropharyngeal swabs and rectal/cloacal swabs), resulting in a total of 3404 processed samples (Figure 2, Supplementary Table S1. Thus, of the 2028 screened individuals, 172 were tested exclusively by ELISA (four positives), 481 exclusively by qPCR (41 positives), and 1375 individuals were tested using both methods (47 positives by ELISA, 97 positives by qPCR, and three positive individuals for both techniques).

Detailed information by species, detection technique, and location are shown in Table 2. The positive individuals by qPCR were differentiated according to species and taxonomic group as follows: 13.4% (127/949) of birds, 2.9% (11/384) of mammals, and no virus was detected in the group of reptiles (0/523) (Supplementary Table S2). The ELISA technique identified seropositive individuals in 5.7% (36/636) of birds, 2.7% (12/446) of mammals, and 1.3% (6/465) of reptiles.

In the group of birds with qPCR screening, the genetic material of Avian Influenza Virus (AIV) was mainly detected in domestic species such as chicken (*Gallus gallus domesticus*, 27.8%, 20/72) and the helmeted guineafowl (*Numida meleagris*, 9.1%, 1/11). Positive samples were also found in wild species such as the wattled jacana (*Jacana jacana*, 18.7%, 14/75), the fork-tailed flycatcher (*Tyrannus savana*, 29.0%, 9/31), the common pauraque (*Nyctidromus albicollis*, 16.1%, 9/56) and the blue-winged teal (*Spatula discors*, 19.3%, 6/31). Serological analysis revealed antibodies to AIV in wild species such as the blue-winged teal (*Spatula discors,* 64.5%, 20/31), the black-bellied whistling duck (*Dendrocygna autumnalis*, 16.7%, 8/48), the orinoco goose (*Oressochen jubatus*, 25%, 7/28) and the tropical screech owl (*Megascops choliba*, 50%, 1/2).

For the mammalian group, as in birds, the highest detection frequency of viral RNA was detected in domestic species, such as horses (*Equus ferus caballus,* 6.4%, 3/47) and cattle (*Bos taurus*, 7.4%, 2/27). The virus was also detected in wild species common in the region, such as the capybara (*Hydrochoerus hydrochaeris,* 1.5%, 2/129) and the armadillo (*Dasypus sabanicola,* 11.8%, 2/17). ELISA results for this taxonomic group showed seropositivity in domestic species such as horses (8.9%, 5/56) and cattle (2.1%, 1/46) and wild species such as capybara (1.9%, 2/107) and lowland paca (*Cuniculus paca,* 100%, 1/1). Antibodies were also detected in bat species such as the flat-faced fruit-eating bat (*Artibeus planirostris*, 6.7%, 1/15), the dark fruit-eating bat (*Artibeus obscurus*, 20%, 1/5), and the pale spear-nosed bat (*Phyllostomus discolor*, 50%, 1/2). In mammals, Influenza A virus, commonly associated with *Sus scrofa* [41,42] was, however, not detected in captured individuals of this species. In contrast to the other taxonomic groups, the results obtained for the reptile group were negative for the detection of IAV RNA by qPCR, whereas the ELISA technique allowed the identification of IAV antibodies in the common caiman (*Caiman crocodilus,* 2.9%, 5/171) and the green iguana (*Iguana iguana,* 2.0% 1/49), which is particularly interesting given the scarcity of reports on the circulation of this pathogen in reptiles.

According to the study locality, we found that in the municipality of Paz de Ariporo, 8.7% (94/1075) of the individuals were positive for IAV. Positivity was notable in some mammals, such as the capybara and the armadillo, and particularly in birds, with 9.4% (46/488) testing positive by qPCR and 11.2% (36/321) by ELISA. The positive bird species belonged mainly to the orders Anseriformes, Galliformes, and Passeriformes. In contrast, in the municipality of El Yopal, 9.7% (95/953) of the animals sampled tested positive. Here, a slightly lower number of birds from the order Anseriformes was captured, as reflected by the absence of positivity in this group (0/315) by ELISA. However, positivity remained notable by qPCR screening, with 17.5% (81/462). The primary tested positive species were tyrant flycatchers, including the fork-tailed flycatcher (*Tyrannus savana*) and the tropical kingbird (*T. melancholicus*).

Finally, the search for previous reports using ELISA or qPCR on the 173 species analyzed in this study revealed that, out of a total of 340 articles published in indexed journals matching the search parameters, 124 species had never been studied as carriers of influenza A virus, five had previously been analyzed for influenza A detection but yielded negative results, and 44 individuals were reported positive for influenza A. A graphical summary of this information is provided in Supplementary Table S3. Additionally, negative results, including the absence of both viral and antibody detection, along with the corresponding number of individuals screened, are listed in Supplementary Table S2.

## 4. Discussion

**Figure 3:**
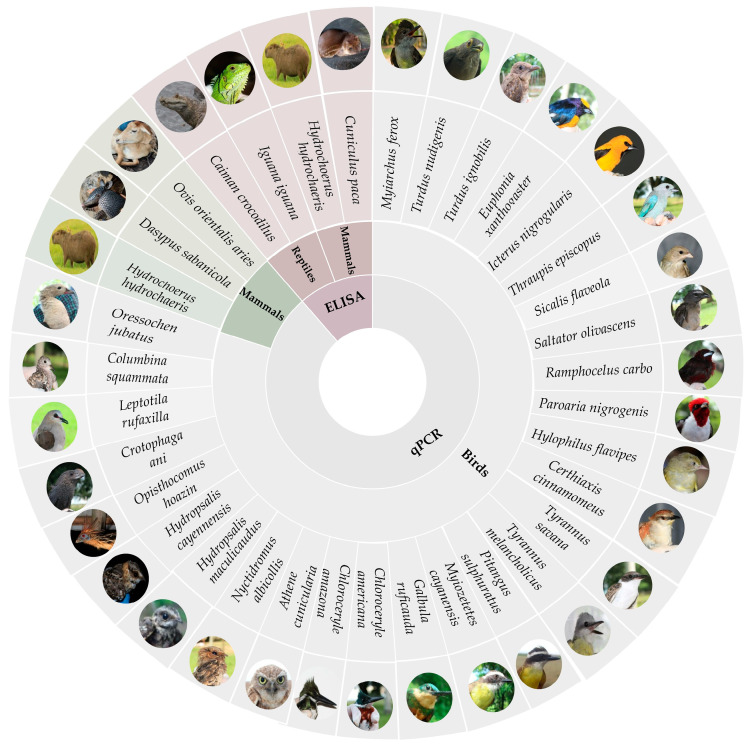
New reports of Influenza A virus detected by ELISA and qPCR in wildlife from the Colombian Orinoco. The figure shows birds, mammals and reptiles in which serological or molecular evidence of the virus was recorded for the first time. The only species commonly detected by both methods was the mammal *Hydrochoerus hydrochaeris*. qPCR did not detect the virus in reptiles, whereas ELISA gave positive results in *Caiman crocodilus* and *Iguana iguana*.

Recent pandemic events have underscored the importance of analyses conducted at broad geographic scales [43], particularly in biodiverse regions such as the Neotropics, where comprehensive data remain scarce. Our results, based on a broad screening in the Orinoco region by molecular and serological methods, revealed a high number of species analyzed and reported as positive for IAV for the first time, that is, 34 species represented in 28 birds, two reptiles, and four mammals (Figure 3). These species are of particular interest because they may represent key hosts as reservoirs or amplification of the virus. On the other hand, our survey included 124 vertebrate species that have never been analyzed for IAV. This is in the context of the approximately 1400 vertebrate species recorded in the Colombian Orinoco [44], which demonstrates the magnitude of the work that remains to be carried out.

In summary, 43 species of birds tested positive out of the 125 sampled over two years, two climatic seasons, and six different locations. Specifically, in the Anseriformes group, 16 qPCR-positive individuals were identified out of 158 (10.1%) and 35 individuals tested positive for ELISA out of 157 (22.3%), suggesting the circulation of the virus in the region or previous exposure. Wild birds of the order Anseriformes are particularly known to be important reservoirs of avian influenza viruses, and their seasonal migrations in search of food resources, favorable climatic conditions, and reproduction facilitate the spread of these viruses [45,46]. According to Afanador-Villamizar [30], in Latin America, approximately 43.7% of reported cases of AIV are attributed to migratory birds, mainly those using established routes from the northern to the southern regions [30]. This phenomenon highlights the importance of wetlands in the Colombian Orinoco, which are essential habitats for migratory birds as stop-overs on their routes [47,48,49]. The particular ecological interactions between migratory and resident species potentially promote the spread of the avian influenza virus in unusual hosts, thus promoting the emergence of new reservoirs, as has been reported in other regions of the world [50,51,52]. Remarkably, in Anseriformes we detected only three positives of the 1375 individuals tested for both ELISA and qPCR: two individuals of the blue-winged teal (*Spatula discors*), a north-to-south migratory, and one orinoco goose (*Oressochen jubatus*), a resident, all recorded in the locality of Paz de Ariporo.

In addition to ducks and geese, the flooded savannas of the Orinoquia region are home to a significant diversity of aquatic and semi-aquatic birds. Of particular interest are species from the heron family (Ardeidae), rail family (Rallidae), plover family (Charadriidae), kingfisher family (Alcedinidae), and hoazin family (Opisthocomidae), which occupy diverse habitats characteristic of these flood-prone environments, including morichales (*Mauritia* palm groves). Among these groups, it is noteworthy that kingfishers and hoatzin exhibited positive results for IAV, while species from the remaining groups exhibited negative results using qPCR and ELISA methods. Although the recurrent entry of IAV by migratory birds, as in the case of waterfowl and waders, can be assumed, the exact mode of transmission and circulation of the virus in resident bird populations remains unclear. It seems reasonable to posit that the initial transmission of the virus occurs horizontally, from infected migrants to resident wild populations with whom they share space, particularly during the dry season, when waterfowl are confined to perennial water bodies and experience high levels of crowding such as in kingfishers and hoatzins [10,11]. Nevertheless, the diverse taxonomic composition of the region may potentially result in a dilution effect by reducing the likelihood of a random encounter with a suitable host, suggesting that a dilution effect rather than an amplification of infection is more likely to occur in the face of high regional biodiversity and diverse functional ecology traits on potential hosts [53,54].

In the floodable savannas of the Orinoquia region, birds share shallow and calm aquatic habitats with other animal groups, making the direct contact and the fecal–oral route plausible mechanisms for the transmission of Influenza A virus [55,56]. Considering this scenario, our study reports, for the first time, the presence of Influenza A virus in the capybara, a species of significant relevance due to its wide distribution across South American wetlands and close contact with humans due to its use as bushmeat. This finding is supported by the two detection approaches implemented, with two individuals testing positive by qPCR and two by ELISA in the locality of Paz de Ariporo. In line with this scenario, reptiles are among the vertebrate groups with limited records of influenza A virus infections. The ability of Influenza A virus to infect and replicate in cell lines in reptiles has been exposed by Temple et al. [57] in the American alligator (*Alligator mississippiensis*), as well as the presence of the virus in certain crocodilian species (*Alligator sinensis*, *Paleosuchus trigonatus*, *Caiman latirostris*, and *Crocodylus niloticus*) held in captivity [21]. In the present study, we report, for the first time, the capacity of Influenza A virus to infect the common caiman, demonstrated by the seropositivity of five individuals: four from the locality of El Yopal (during both dry and wet seasons) and one from Paz de Ariporo (dry season); plus, one case for the green iguana in El Yopal (Dry season). Although the diagnostic kit used has primarily been validated for birds and mammals, our results in reptiles highlight the importance of screening this taxonomic group and, together with the new report for capybaras, raise new questions regarding the relevance of wild species as potential accidental hosts in the virus transmission cycle.

It is noteworthy that doves, cuckoos, nightjars, vultures, and jacamars, in addition to songbirds such as tyrant flycatchers, ovenbirds, vireos, and tanagers, also registered IAV positivity by qPCR. Conversely, in such instances, the former aquatic ecological factors are not readily discernible, thereby rendering it more challenging to ascertain the potential interrelationships between waterfowl and the aforementioned avian taxa. It is worth mentioning the tyrant flycatchers, exhibiting a notable positivity rate of 13.5% (19/141), as well as the fork-tailed flycatcher (*Tyrannus savana*), an austral migratory species, with a positivity rate of 29% (9/31). Similarly, the tropical kingbird (*Tyrannus melancholicus*), also belonging to the genus *Tyrannus*, with 13% (4/30), demonstrated a notable positivity rate. Remarkably, these species coexist during the humid season, and both can inhabit transformed environments, including those resulting from land expansion and human population density [15]. High-intensity agricultural practices in the Orinoquia, such as cattle raising, rice and oil palm crops [58,59] may force migratory birds to interact more closely with other species, thereby facilitating the spread of AIV within these transformed ecosystems [60]. Consistently, ecosystem conservation is associated with the protection of functional complexity, and thus with the dilution effect, since the degradation of wetlands supporting species has been associated with a lower diversity of ecological functions and with a higher potential risk of transmission of pathogens such as IAV [61]. Hence, such species associated with transformed environments represent priority targets for future surveillance of this pathogen in the region.

Again, thanks to the extensive sampling effort and diversity of the region, our study reports influenza A virus circulation in bats, with seropositive samples detected in spear-nosed bats *Phyllostomus discolor* with 50% (1/2), in the dark fruit bat *Artibeus obscurus* with 20% (1/5), and flat-faced fruit bats *Artibeus planirostris* with 5.5% (1/18). These results are consistent with previous studies that have reported serologic reactivity within the genus, including the Jamaican fruit (*Artibeus jamaicensis*), the great fruit-eating bat (*A. lituratus*), the pygmy fruit-eating bat (*A. phaeotis*), and dusky (*A. phaeotis*) and the dark fruit bat in Guatemala, as well as the dark fruit bat, the flat-faced fruit bat, and the great fruit-eating bat in Peru [33]. In particular, our results agree with previous reports of IAV in Casanare for the spear-nosed bat [62].

We also detected positivity in livestock, which, although not the primary target of the study, may provide insights into the dynamics of virus circulation in the region. Notably, chicken (*Gallus gallus domesticus*) was the species with the highest number of positive individuals, and during both season periods, with 27.8% (20/72), all detected by qPCR. Horses (*Equus ferus caballus*)*,* with 5.3% (3/56) by qPCR and 8,9% (5/56) by ELISA and cattle (*Bos taurus*), with 4.2% (2/47) by qPCR and 2,2% (1/46) by ELISA; were the only two livestock species detected by both techniques. Remarkably, positive detection in domestic sheep (*Ovis orientalis aries domestica*), with 16.7% (2/12) positives by qPCR and none by ELISA, represents the first global report of IAV presence, according to our search results (Supplementary Table S1). These findings are particularly interesting, as the livestock sampled are maintained under free-grazing practices, facilitating interactions with wildlife, a favorable scenario for IAV propagation [41,63] and are consistent with previous reports from the Colombian Orinoquia, which documented positivity in poultry with 2.8% (31/1188) and 4.1% for horses (9/220) [64]. In contrast to those findings, where positivity was reported in *Sus scrofa domestica* with 13.4% (91/678), we did not detect the presence of the virus in either farm-raised (n = 9) or wild (n = 48) individuals. The avian-to-swine host jump is a topic of particular importance in influenza epidemiology, as it has direct health implications for swine [65] and their role as intermediaries in the adaptation of avian viruses to humans [66]. Overall, our results highlight the importance of constant monitoring for disease, as undetected infections could have cascading effects on the overall health of livestock populations, particularly in regions where these industries are economically significant, such as the Orinoquia [67].

## 5. Conclusions

Our study makes a significant contribution to determining potential reservoirs or incidental hosts in the IAV transmission cycle in the Orinoquia region of Colombia, revealing 54 positive species, 34 of which represent the first recorded cases of IAV worldwide. These results emphasize the need to expand research to less explored taxa in wildlife and their ecological interactions with migratory and resident birds, as well as the impact of anthropized environments as key factors in the transmission of the avian influenza virus.

In this context, the need for an ongoing wildlife surveillance and monitoring system becomes evident, particularly considering recent increases in infection rates among various mammal species due to influenza A virus. This system should prioritize the characterization of virus variants and monitor viral flow directions between migratory and resident wildlife. Two crucial methodological recommendations are highlighted to improve in situ wildlife studies: first, access to specialized infrastructure for viral culture; and second, proper maintenance of the cold chain, especially in rural areas, where challenges such as deficiencies in the electrical grid and adverse climatic conditions compromise viral genome stability. These improvements are essential for enhancing detection sensitivity and genomic data quality for future characterization.

## Figures and Tables

**Table 1:** Individuals screened and positive detections by each technique from each taxonomic group in the two localities and seasons (2023–2024). n = total number of individuals screened.

		BIRDS				MAMMALS				REPTILES			
		ELISA		qPCR		ELISA		qPCR		ELISA		qPCR	
		n	Positives	n	Positives	n	Positives	n	Positives	n	Positives	n	Positives
Rainy	PazAriporo	157	34	177	17	70	0	91	0	68	0	79	0
	El Yopal	57	0	111	13	52	2	54	0	75	2	99	0
Dry	PazAriporo	164	2	311	29	190	7	183	7	149	1	170	0
	El Yopal	258	0	351	68	134	3	56	4	173	3	175	0
TOTALS		636	36	950	127	446	12	384	11	465	6	523	0

**Table 2:** Number of individuals of each species detected as positive by type of technique, location, order and taxonomic group. n = total number of individuals of the species screened. Asterisks (*) indicate species corresponding to livestock.

		Paz de Ariporo			El Yopal		
		n	qPCR	ELISA	n	qPCR	ELISA
Birds	Order Anseriformes						
	*Amazonetta brasiliensis*	4	3	0	2	0	0
	*Cairina moschata*	19	4	0	15	0	0
	*Dendrocygna autumnalis*	44	0	8	5	0	0
	*Oressochen jubatus*	25	3	7	3	0	0
	*Spatula discors*	31	6	20	0	0	0
	Order Pelecaniformes						
	*Ardea cocoi*	0	0	0	1	1	0
	*Platalea ajaja*	16	2	0	0	0	0
	Order Gruiformes						
	*Porphyrio martinica*	0	0	0	1	1	0
	Order Galliformes						
	*Gallus gallus* *	27	8	0	45	12	0
	*Numida meleagris* *	4	1	0	7	0	0
	Order Columbiformes						
	*Columbina squammata*	5	0	0	7	1	0
	*Leptotila rufaxilla*	8	1	0	4	0	0
	Order Cuculiformes						
	*Crotophaga ani*	0	0	0	15	4	0
	Order Opisthocomiformes						
	*Opisthocomus hoazin*	0	0	0	1	1	0
	Order Charadriiformes						
	*Himantopus mexicanus*	19	1	0	2	0	0
	*Jacana jacana*	9	0	0	66	14	0
	*Hoploxypterus cayanus*	7	1	0	1	0	0
	*Vanellus chilensis*	4	1	0	11	1	0
	Order Strisores						
	*Hydropsalis cayennensis*	0	0	0	13	3	0
	*Hydropsalis maculicaudus*	0	0	0	2	1	0
	*Nyctidromus albicollis*	17	2	0	39	7	0
	Order Strigiformes						
	*Athene cunicularia*	3	0	0	9	2	0
	*Megascops choliba*	1	0	1	1	0	0
	Order Accipitriformes						
	*Cathartes aura*	2	1	0	0	0	0
	Order Coraciiformes						
	*Chloroceryle amazona*	2	0	0	3	3	0
	*Chloroceryle americana*	8	1	0	2	0	0
	Order Piciformes						
	*Galbula ruficauda*	0	0	0	2	1	0
	Order Passeriformes						
	*Myiarchus ferox*	4	1	0	2	1	0
	*Myiozetetes cayanensis*	4	0	0	11	2	0
	*Pitangus sulphuratus*	11	1	0	10	1	0
	*Tyrannus melancholicus*	10	0	0	20	4	0
	*Tyrannus savana*	0	0	0	31	9	0
	*Certhiaxis cinnamomeus*	0	0	0	1	1	0
	*Hylophilus flavipes*	2	2	0	0	0	0
	*Paroaria nigrogenis*	8	0	0	7	3	0
	*Ramphocelus carbo*	7	1	0	4	1	0
	*Saltator olivascens*	2	1	0	3	1	0
	*Sicalis flaveola*	8	0	0	4	1	0
	*Thraupis episcopus*	6	1	0	7	2	0
	*Icterus nigrogularis*	2	0	0	4	1	0
	*Euphonia xanthogaster*	0	0	0	1	1	0
	*Turdus ignobilis*	4	2	0	5	1	0
	*Turdus nudigenis*	10	2	0	0	0	0
Mammals	Order Artiodactyla						
	*Ovis orientalis aries* *	12	2	0	0	0	0
	*Bos taurus* *	15	0	0	32	2	1
	Order Chiroptera						
	*Artibeus obscurus*	1	0	0	4	0	1
	*Artibeus planirostris*	5	0	0	11	0	1
	*Phyllostomus discolor*	2	0	1	0	0	0
	Order Cingulata						
	*Dasypus sabanicola*	17	2	0	1	0	0
	Order Perissodactyla						
	*Equus ferus caballus* *	18	1	4	38	2	1
	Order Rodentia						
	*Hydrochoerus hydrochaeris*	126	2	2	8	0	0
	*Cuniculus paca*	0	0	0	3	0	1
Reptiles	Order Squamata						
	*Iguana iguana*	21	0	0	44	0	1
	Order Crocodylia						
	*Caiman crocodilus*	100	0	1	90	0	4

## Data Availability

The original contributions presented in this study are included in Supplementary Table S1 Raw data screening.

## Supplementary Materials

The following supporting information can be downloaded at https://www.mdpi.com/article/10.3390/ani15152201/s1, Table S1: Raw data screening; Table S2: Number of individuals per species of birds, mammals, and reptiles screened and negative; Table S3: Status of previous reports for Influenza A virus in the sampled species by ELISA or qPCR.

## Abbreviations

The following abbreviations are used in this manuscript:

DOAJ	Directory of open access journals
IAV	Influenza A Virus
AIV	Avian Influenza Virus
qPCR	Quantitative Polymerase Chain Reaction
ELISA	Enzyme Linked Immunosorbent Essay
CT	Cycle Threshold
