# Supplementary material for: A Broad Wildlife Survey of Influenza A Virus in the Orinoco Flooded Savannas from Colombia: New Reports and Perspectives
Source: Animals (Basel). 2025 Jul 26;15(15):2201. doi: 10.3390/ani15152201 (PMC12345564; doi:10.3390/ani15152201)
Supplement: Supplementary file 1 [file animals-15-02201-s001.zip › Supplementary Table S2 Number of individuals per species of birds, mammals and reptiles screened and negative results for the Influenza A virus by qPCR or ELISA test in Paz de Ariporo and El Yopal C.PDF]

Supplementary Table S2: Number of individuals per species of birds, mammals and reptiles screened and negative results for the Influenza A virus by qPCR or ELISA test in Paz de Ariporo and El Yopal Casanare, Colombia.

		Paz de Ariporo			El Yopal		
		**n**	qPCR	ELISA	n	qPCR	ELISA
	**Family Accipiteridae**						
	Buteo albonotatus	1	1	1	**1**	0	0
	**Family Accipitridae**						
	Rupornis magnirostris	1	0	0	1	1	1
	**Family Alcedinidae**						
	Chloroceryle aenea	3	2	2	3	1	1
	Chloroceryle amazona	2	2	2	3	0	0
	Chloroceryle americana	8	7	8	2	2	3
	Megaceryle torquata	1	1	1	2	2	2
	**Family Anatidae**						
	Amazonetta brasiliensis	10	7	10	2	2	2
	Anser anser	1	1	1	2	2	2
	Cairina moschata	19	15	19	1	1	1
	Dendrocygna autumnalis	44	44	36	1	5	5
	Dendrocygna viduata	0			1	1	1
	Oressochen jubatus	23	22	18	2	2	2
	Spatula discors	31	25	11	0		
	**Family Anhingidae**						
	Anhinga anhinga	1	1	1	1		
	**Family Ardeidae**						
	Ardea cocoi	0	**0**	0	1	**0**	1
	Bubulcus ibis	7	7	7	2	2	2
	Butorides striata	0	0	0	1	1	1
**Birds**	*Egretta caerulea*	6	4	4	6	2	2
	Egretta thula	2	2	2	2	2	2
	Tigrisoma lineatum	0			1	1	1
	**Family Burhinidae**						
	Hesperoburhinus bistriatus	1	1	1	0		
	**Family Caprimulgidae**						
	Chordeiles nacunda	2	2	2	2	2	2
	Hydropsalis cayennensis	2	2	2	13	10	13
	Hydropsalis maculicaudus	1	1	1	2	1	2
	Jabiru mycteria	1	1	1	0	0	0
	Myiozetetes cayanensis	2	2	2	0	0	0
	Nyctidromus albicollis	17	15	17	39	32	39
	Nyctiprogne leucopyga	1	1	1	1	1	1
	**Family Cardinalidae**						
	Saltator coerulescens	4	2	2	2	2	2
	Saltator maximus	2			2	2	2
	**Family Cathartidae**						

Cathartes aura	1	1	2	2		
Coragyps atratus	6	4	4	6	2	2
Sarcoramphus papa	1			1	1	1
**Family Charadriidae**						
Anarhynchus collaris	1	1	1	0		
Hoploxypterus cayanus	7	6	7	1	1	1
Vanellus chilensis	4	3	4	11	10	11
**Family Ciconiidae**						
Eudocimus ruber	1	1	1	0		
Mycteria americana	3	3	3	0		
**Family Columbidae**						
Columbina minuta	5	5	5	9	9	9
Columbina squammata	5	5	5	7	6	7
Columbina talpacoti	0			1	1	1
Leptotila rufaxilla	8	7	8	4	4	4
Leptotila verreauxi	2	2	2	9	9	9
Patagioenas cayennensis	1	1	1	1	1	1
**Family Cracidae**						
Ortalis ruficauda	0			1	1	1
**Family Cuculidae**						
Coccycua minuta	1					
Crotophaga ani	0	0	0	15	11	15
Crotophaga major	0			2	2	2
**Family Emberizidae**						
Ammodramus aurifrons	0			1	1	1
**Family Eurypygidae**						
Eurypyga helias	3	3	3	0		
**Family Falconidae**	1	**1**				
Milvago chimachima	1	1	1	0		
Euphonia xanthogaster	0			1		1
**Family Furnariidae**						
Certhiaxis cinnamomeus	0			1	1	1
Cranioleuca vulpina	0	0	0	1	1	1
Dendroplex picus	1	1	1	1	1	1
Phacellodomus rufifrons	4	4	4	3	3	3
Synallaxis albescens	0			1	**1**	1
**Family Galbulidae**						
Galbula ruficauda	0	**0**	0	2	**2**	2
**Family Icteridae**						
Cacicus cela	4	4	4	0		
Gymnomystax mexicanus	4	4	4	0	2	
Icterus icterus	0	0		2	3	2
Icterus nigrogularis	2	2	2	4	3	4
Leistes militaris	0	0	0	1	1	1

Quiscalus lugubris	10	10	10	0		
Sturnella magna	1	**1**	1	1	**1**	1
**Family Jacanidae**						
Jacana jacana	9	9	9	66	52	66
**Family Mimidae**						
Mimus gilvus	0	**0**		7	**7**	7
**Family Numididae**						
Numida meleagris	4	**3**	4	7	**7**	7
**Family Nyctibiidae**						
Nyctibius grandis	1			3	**1**	3
**Family Odontophoridae**						
Colinus cristatus	0	**0**	0	1	**1**	1
**Family Phalacrocoracidae**						
Phalacrocorax brasilianus	2	**2**	2	0	**0**	0
**Family Phasianidae**						
Gallus gallus	27	15	27	45	38	45
Meleagris gallopavo	0	0	0	3	1	3
Pavo cristatus	0	**0**	0	1	**1**	1
**Family Phasianidae_**						
**Picidae**						
Colaptes punctigula	0	0	0	1	1	1
Picumnus squamulatus	1	**1**	1	1	**1**	1
**Family Psittacidae**						
Ara macao	0	0	0	1	1	1
Forpus conspicillatus	*0*	**0**	0	3	**3**	3
**Family Rallidae**						
Aramides cajaneus	5	5	5	1	1	1
Phorphirio flavirostris	0	0	0	2	2	2
Porphyrio martinica	0			1	**0**	1
**Family Recurvirostridae**						
Himantopus mexicanus	19	**18**	19	2	**2**	2
**Family Scolopacidae**						
Calidris minutilla	2	2	2	0	0	0
Tringa solitaria	2	**2**	2	0	**0**	0
**Family Strigidae**						
Athene cunicularia	3	3	3	9	7	9
Megascops choliba	1	**1**	0	1	**1**	1
**Family Thamnophilidae**						
Formicivora grisea	1	1	1	1	1	1
Thamnophilus doliatus	4	4	4	0	**0**	0
**Family Thraupidae**						
Coereba flaveola	0			2	2	2
Paroaria nigrogenis	8	8	8	7	4	7
Ramphocelus carbo	7	6	7	4	3	4

Saltator olivascens	2	1	2	3	2	3
Sicalis flaveola	8	8	8	4	3	4
Sporophila intermedia	4	4	4	1	1	1
Stilpnia cayana	1	1	1	2	2	2
Tachyphonus rufus	2	2	2	0	0	0
Thraupis episcopus	6	5	6	7	5	7
Thraupis palmarum	1	1	1	1	1	1
**Family Threskiornithidae**						
Eudocimus ruber	5	5	5	0	0	0
Phimosus infuscatus	2	2	2	1	1	1
Platalea ajaja	16	14	16	0	0	0
Theristicus caudatus	1	1	1	0		
**Family Turdidae**						
Turdus ignobilis	4	2	4	5	4	5
Turdus leucomelas	2	2	2	0		
Turdus nudigenis	10	8	10	0	0	0
**Family Tyrannidae**						
Elaenia chiriquensis	4	4	4	1	1	1
Elaenia flavogaster	1	1	1	2	2	2
Elaenia parvirostris	5	5	5	2	2	2
Fluvicola pica	1	1	1	1	1	1
Megarynchus pitangua	2	2	2	0	0	0
Myiarchus cephalotes	5	5	5	0	0	0
Myiarchus ferox	4	3	4	2	1	2
Myiarchus swainsoni	0	0	0	1	1	1
Myiarchus tyrannulus	1	1	1	1	1	1
Myiopagis gaimardii	1	1	1	0	0	0
Myiozetetes cayanensis	4	4	4	11	9	11
Phelpsia inornata	5	5	5	1	1	1
Philohydor lictor	2	2	2	0	0	0
Pitangus sulphuratus	11	10	11	10	9	10
Rhytipterna simplex	1	1	1	0		
Tyrannus melancholicus	10	10	10	20	16	20
Tyrannus savana	0			31	22	31
**Family Vireonidae**						
Cyclarhis gujanensis	1	1	1	0		
Hylophilus flavipes	2	0	2	0	0	0
**Family Atelidae**						
Alouatta seniculus	0			1	1	1
**Family Bovidae**						
Bos taurus	15	15	15	32	30	31
Bubalus bubalis	2	2	2	0	0	0
Capra aegagrus hircus	0	0	0	4	4	4
ovis orientalis aries domestica	12	10	12	0		

	**Family Canidae**						
	Cerdocyon thous	1	1	1	0		
	**Family Caviidae**						
	Hydrochoerus hydrochaeris	126	124	124	8	8	8
	**Family Cuniculidae**						
	Cuniculus paca	0			3	3	2
	**Family Dasypodidae**						
	Dasypus sabanicola	16	15	17	18	1	1
	**Family Dasyproctidae**						
	Dasyprocta fuliginosa	12	12	12	0		
**Mammals**	**Family Didelphidae**						
	Didelphis marsupialis	10	10	10	20	20	20
	Caluromys sp.	1	0	0	0	0	0
	**Family Echimyidae**						
	Proechimys oconnelli	1	1	1	0	0	0
	Proechimys semispinosus	0	0	0	1	1	1
	**Family Emballonuridae**						
	Saccopteryx leptura	2	2	2	0	0	0
	**Family Equidae**						
	Equus ferus caballus	18	17	14	38	36	37
	**Family Molossidae**						
	Eumops glaucinus	0	0	0	2	2	2
	Molossus molossus	0	0	0	8	8	8
	Molossus pretiosus	10	10	10	4	4	4
	**Family Myrmecophagidae**						
	Myrmecophaga tridactyla	5	5	5	13	13	13
	Tamandua tetradactyla	0	**0**	0	5	**5**	5
	**Family Noctilionidae**						
	Noctilio albiventris	6	6	6	5	5	5
	**Family Phyllostomidae**						
	Sturnira giannae	1	1	1	0	0	0
	Artibeus lituratus	1	0	0	0	0	0
	Artibeus obscurus	1	1	1	3	3	2
	Artibeus planirostris	4		8	17	4	9
	Carollia brevicauda	0	0	0	3	3	3
	Carollia perspicillata	2	2	2	6	6	6
	Dermanura gnoma	0	0	0	1	1	1
	Desmodus rotundus	0	0	0	1	1	1
	Lonchophylla orcesi	0	0	0	1	1	
	Lophostoma brasiliense	0	0	0	2	2	1
	Micronycteris schmidtorum	0	0	0	2	1	2
	Phyllostomus discolor	2	2	1	0	0	0
	Phyllostomus hastatus	0	0	0	1	1	1
	Trachops cirrhosus	1	1	1	4	4	3

Uroderma bilobatum	1	**1**	1	1	**1**	1
Uroderma magnirostrum	0	**0**	0	2	**2**	2
**Family Suidae**						
Sus scrofa	46	46	46	6	6	6
**Family Vespertilionidae**						
Myotis nigricans	1	1	1	3	3	3
Myotis riparius	1	1	1	2	2	2
Rhogeessa io	0	0	0	1	1	1
**Family Leporidae**						
Oyctolagus cuniculus	0	0	0	2	2	2
**Family Teiidae**						
Tupinambis teguixin	11	11	11	8	8	8
**Family Testudinidae**						
Chelonoidys carbonara	14	14	14	39	39	39
**Family Alligatoridae**						
Caiman crocodilus	100	100	99	90	90	86
**Family Boidae**						
Eunectes murinus	0	0	0	2	2	2
**Family Iguanidae**						
Iguana iguana	20	20	20	44	44	43
**Family Podocnemididae**						
Podocnemis vogli	125	**125**	125	106	**106**	106
