# Supplementary material for: A Broad Wildlife Survey of Influenza A Virus in the Orinoco Flooded Savannas from Colombia: New Reports and Perspectives
Source: Animals (Basel). 2025 Jul 26;15(15):2201. doi: 10.3390/ani15152201 (PMC12345564; doi:10.3390/ani15152201)
Supplement: Supplementary file 1 [file animals-15-02201-s001.zip › Supplementary Table S3_ Status of previous reports for Influenza A virus in the sampled species by ELISA or qPCR.pdf]

Supplementary Table S3: Status of previous reports for Influenza A virus in the sampled species by ELISA or qPCR

Scientific name	Common name	Status
Amazonetta brasiliensis	Brazilian Teal	IAv positive in previous studies
Ammodramus aurifrons	Yellow-browed Sparrow	No previous reports
Anarhynchus collaris	Wrybill	No previous reports
Anhinga anhinga	Anhinga (or Snakebird)	No previous reports
Anser anser	Greylag Goose	IAv positive in previous studies
Ara macao	Scarlet Macaw	No previous reports
Aramides cajaneus	Gray-cowled Wood-Rail	No previous reports
Ardea cocoi	Garza cocoi	IAv positive in previous studies
Athene cunicularia	Burrowing Owl	No previous reports
Bubulcus ibis	Cattle Egret	No previous reports
Buteo albonotatus	Zone-tailed Hawk	No previous reports
Butorides striata	Striated Heron	No previous reports
Cacicus cela	Yellow-rumped Cacique	No previous reports
Cairina moschata	Muscovy duck	IAv positive in previous studies
Calidris minutilla	Least Sandpiper	IAv positive in previous studies
Cathartes aura	turkey vulture	IAv positive in previous studies
Certhiaxis cinnamomeus	Yellow-chinned Spinetail	No previous reports
Chloroceryle aenea	American Pygmy Kingfisher	No previous reports
Chloroceryle amazona	Amazon Kingfisher	No previous reports
Chloroceryle americana	Green Kingfisher	No previous reports
Chordeiles nacunda	Nacunda Nighthawk	No previous reports
Coccycua minuta	Little Cuckoo	No previous reports
Coereba flaveola	Bananaquit	No previous reports
Colaptes punctigula	Spot-breasted Woodpecker	No previous reports
Colinus cristatus	Crested Bobwhite	No previous reports
Columbina minuta	Plain-breasted Ground-Dove	No previous reports
Columbina squammata	Scaled Dove	No previous reports
Columbina talpacoti	Ruddy Ground-Dove	IAv positive in previous studies
Coragyps atratus	Black Vulture	IAv positive in previous studies
Cranioleuca vulpina	Buff-throated Foliage-gleaner	No previous reports
Crotophaga ani	Smooth-billed Ani	No previous reports
Crotophaga major	Greater Ani	No previous reports
Cyclarhis gujanensis	Rufous-browed Peppershrike	No previous reports
Dendrocygna autumnalis	Dendrocygna autumnalis	IAv positive in previous studies
Dendrocygna viduata	White-faced Whistling-Duck	IAv positive in previous studies
Dendroplex picus	Straight-billed Woodcreeper	No previous reports
Dryobates fumigatus	Smoky-brown Woodpecker.	No previous reports
Egretta caerulea	Little Blue Heron	IAv negative in previous studies
Egretta thula	Snowy Egret	IAv positive in previous studies
Elaenia chiriquensis	Lesser Elaenia	No previous reports
Elaenia flavogaster	Yellow-bellied Elaenia	No previous reports
Elaenia parvirostris	Small-billed Elaenia	No previous reports

	Eudocimus ruber	Scarlet Ibis	IAv positive in previous studies
	Euphonia xanthogaster	Orange-bellied Euphonia	No previous reports
	Eurypyga helias	Sunbittern	No previous reports
	Fluvicola pica	Pied Water-Tyrant	No previous reports
	Formicivora grisea	White-fringed Antwren	No previous reports
	Forpus conspicillatus	Spectacled Parrotlet	No previous reports
	Galbula ruficauda	Rufous-tailed Jacamar	No previous reports
	Gallus gallus	chicken	IAv positive in previous studies
	Gymnomystax mexicanus	Oriole Blackbird	No previous reports
	Hesperoburhinus bistriatus	Double-striped Thick-knee	No previous reports
	Himantopus mexicanus	helmeted guineafowl	IAv positive in previous studies
	Hoploxypterus cayanus	Pied plover	No previous reports
	Hydropsalis cayennensis	White-tailed Nightjar	No previous reports
	Hydropsalis maculicaudus	Spot-tailed Nightjar	No previous reports
	Hylophilus flavipes	Scrub Greenlet	No previous reports
	Icterus icterus	brilletrupial	No previous reports
	Icterus nigrogularis	Yellow Oriole	No previous reports
	Jabiru mycteria	Jabiru	No previous reports
	Jacana jacana	wattled jacana	IAv positive in previous studies
	Leistes militaris	Red-breasted meadowlark	No previous reports
**Birds**	*Leptotila rufaxilla*	Grey-fronted Dove	IAv negative in previous studies
	Leptotila verreauxi	White-tipped Dove	No previous reports
	Megaceryle torquata	Ringed Kingfisher	No previous reports
	Megarynchus pitangua	Boat-billed Flycatcher	No previous reports
	Megascops choliba	Tropical Screech-Owl	IAv positive in previous studies
	Meleagris gallopavo	Wild Turkey	IAv positive in previous studies
	Milvago chimachima	Yellow-headed Caracara	No previous reports
	Mimus gilvus	Tropical Mockingbird	No previous reports
	Mycteria americana	Wood Stork	IAv positive in previous studies
	Myiarchus cephalotes	Pale-edged Flycatcher	No previous reports
	Myiarchus ferox	Short-crested Flycatcher	No previous reports
	Myiarchus swainsoni	Swainson's Flycatcher	No previous reports
	Myiarchus tyrannulus	Brown-crested Flycatcher	No previous reports
	Myiopagis gaimardii	Forest Elaenia	No previous reports
	Myiozetetes cayanensis	Rusty-margined Flycatcher	No previous reports
	Numida meleagris	Wattled jacana	IAv positive in previous studies
	Nyctibius grandis	Great Potoo	No previous reports
	Nyctidromus albicollis	common pauraque	No previous reports
	Nyctiprogne leucopyga .	Band-tailed Nighthawk	No previous reports
	Opisthocomus hoazin	Hoatzin	No previous reports
	Oressochen jubatus	Orinoco Goose	No previous reports
	Ortalis ruficauda	Rufous-vented Chachalaca	No previous reports
	Paroaria nigrogenis	Masked Cardinal	No previous reports
	Patagioenas cayennensis	Pale-vented Pigeon	No previous reports

Pavo cristatus	Indian Peafowl	IAv positive in previous studies
Phacellodomus rufifrons	Rufous-fronted Thornbird	No previous reports
Phalacrocorax brasilianus	Neotropic Cormorant	IAv positive in previous studies
Phelpsia inornata	Phelps's Widowbird	No previous reports
Philohydor lictor	Lesser Kiskadee	No previous reports
Phimosus infuscatus	Bare-faced Ibis	IAv positive in previous studies
Phorphirio flavirostris	Azure Gallinule	No previous reports
Picumnus squamulatus	Scaled Piculet	No previous reports
Pitangus sulphuratus	Great kiskadee	No previous reports
Platalea ajaja	Roseate spoonbill	IAv positive in previous studies
Porphyrio flavirostris	Azure Gallinule	No previous reports
Porphyrio martinica	Purple gallinule	No previous reports
Quiscalus lugubris	Carib Grackle	No previous reports
Ramphocelus carbo	Silver-beaked Tanager	No previous reports
Rhytipterna simplex	Greyish mourner	No previous reports
Rupornis magnirostris	Roadside hawk	No previous reports
Saltator coerulescens	Bluish-grey saltator	No previous reports
Saltator maximus	Buff-throated saltator	No previous reports
Saltator olivascens	Olive-gray Saltator	No previous reports
Sarcoramphus papa	King vulture	No previous reports
Sicalis flaveola	Saffron Finch	No previous reports
Spatula discors	Blue-winged teal	IAv positive in previous studies
Sporophila intermedia	Gray Seedeater	No previous reports
Stilpnia cayana	Burnished-buff tanager	No previous reports
Sturnella magna	Eastern meadowlark	No previous reports
Synallaxis albescens	Pale-breasted spinetail	No previous reports
Tachyphonus rufus	White-lined tanager	No previous reports
Thamnophilus doliatus	Barred antshrike	No previous reports
Theristicus caudatus	buff-necked ibis	No previous reports
Thraupis episcopus	Blue-gray tanager	No previous reports
Thraupis palmarum	Palm tanager	No previous reports
Tigrisoma lineatum	Rufescent tiger heron	No previous reports
Tringa solitaria	Solitary sandpiper	No previous reports
Turdus ignobilis	Black-billed Thrush	No previous reports
Turdus leucomelas	Pale-breasted thrush	No previous reports
Turdus nudigenis	Bare-eyed Thrush	No previous reports
Tyrannus melancholicus	Tropical Kingbird	No previous reports
Tyrannus savana	Fork-tailed flycatcher	No previous reports
Vanellus chilensis	Southern lapwing	IAv negative in previous studies
Alouatta seniculus	Red Howler Monkey	No previous reports
Artibeus lituratus	Great fruit-eating bat	IAv positive in previous studies
Artibeus obscurus	Dark fruit-eating bat	IAv positive in previous studies
Artibeus planirostris	Flat-faced fruit-eating bat	IAv positive in previous studies
Bos taurus	Cow	IAv positive in previous studies

Mammals	*Bubalus bubalis*	Water Buffalo	IAv positive in previous studies
	Caluromys sp.	Woolly Opossums	No previous reports
	Capra aegagrus hircus	Wild Goat	No previous reports
	Carollia brevicauda	Silky short-tailed bat	No previous reports
	Carollia perspicillata	Seba's short-tailed bat	IAv positive in previous studies
	Cerdocyon thous	Crab-eating Fox	No previous reports
	Cuniculus paca	Lowland Paca	No previous reports
	Dasyprocta fuliginosa	Black Agouti	No previous reports
	Dasypus sabanicola	Llanos Long-nosed Armadillo	No previous reports
	Desmodus rotundus	Common vampire bat	IAv positive in previous studies
	Didelphis marsupialis	Common Opossum	IAv positive in previous studies
	Equus ferus caballus	Horse	IAv positive in previous studies
	Eumops glaucinus	Wagner's bonneted bat	IAv negative in previous studies
	Hydrochoerus hydrochaeris	Capybara	No previous reports
	Lophostoma brasiliense	Pygmy round-eared bat	No previous reports
	Molossus molossus	Velvety free-tailed bat	IAv positive in previous studies
	Molossus pretiosus	Miller's mastiff bat	No previous reports
	Myotis nigricans	Black myotis	IAv positive in previous studies
	Myotis riparius	Riparian myotis	IAv negative in previous studies
	Myrmecophaga tridactyla	Giant Anteater	IAv positive in previous studies
	Noctilio albiventris	Lesser bulldog bat	IAv positive in previous studies
	Oryctolagus cuniculus	European rabbit	IAv positive in previous studies
	Ovis orientalis aries	Domestic Sheep	No previous reports
	Phyllostomus discolor	Pale spear-nosed bat	IAv positive in previous studies
	Phyllostomus elongatus	Lesser spear-nosed bat	No previous reports
	Phyllostomus hastatus	Greater spear-nosed bat	No previous reports
	Proechimys oconnelli	O'Connell's Spiny Rat	No previous reports
	Proechimys sp.	Spiny rats	No previous reports
	Rhogeessa io	Thomas's yellow bat	No previous reports
	Saccopteryx leptura	Lesser sac-winged bat	No previous reports
	Sturnira giannae	Yellow-shouldered	IAv positive in previous studies
	Sus scrofa domestica	Pig	IAv positive in previous studies
	Sus scrofa silvestre	Wild Boar	No previous reports
	Tamandua tetradactyla	Southern Tamandua	No previous reports
	Trachops cirrhosus	Fringe-lipped bat	IAv positive in previous studies
	Uroderma bilobatum	Tent-making bat	IAv positive in previous studies
	Uroderma magnirostrum	Brown tent-making bat	IAv positive in previous studies
Reptil	*Caiman crocodilus*	Caiman	No previous reports
	Chelonoidys carbonara	Red-footed Tortoise	No previous reports
	Eunectes murinus	Green anaconda	No previous reports
	Iguana iguana	Green Iguana	No previous reports
	Podocnemis vogli	Llanos Sideneck Turtle	No previous reports
	Tupinambis teguixin	Gold Tegu	No previous reports
